# Growth Mindset as a Personal Preference Predicts Teachers’ Favorable Evaluation of Positive Education as an Imported Practice When Institutional and Normative Support for It Are Both Strong or Both Weak

**DOI:** 10.3389/fpsyg.2020.00934

**Published:** 2020-06-12

**Authors:** Vincci Chan, Chi-yue Chiu, Sau-lai Lee, Iris Leung, Yuk-Yue Tong

**Affiliations:** Faculty of Social Science, The Chinese University of Hong Kong, Shatin, China

**Keywords:** growth mindset, personal preference, institution, perceived descriptive norm, cultural influence, cultural change

## Abstract

Past research on pathways to cultural influence on judgment has compared the explanatory power of personal preferences, perceived descriptive norms and institutionalization. Positive education is an education movement inspired by Western positive psychology. The present study examined how these factors jointly predict Hong Kong teachers’ evaluation of imported positive education programs in their schools. In a field study, we measured teachers’ personal endorsement of growth mindset (a positive psychology construct developed in the US) and their evaluation of adopting positive education programs in their schools. We also measured teachers’ perception of the extent of institutional and normative support for positive education in their schools. The results show that teachers’ personal preferences for growth mindset predict more favorable evaluation of positive education programs when institutional and normative support for positive education programs are *both* weak, *or* when they are *both* strong. We interpret these effects from the perspectives of the strong situation hypothesis and the intersubjective theory of culture.

## Introduction

“As scholars have noted of positive psychology …, the emphasis on growth and personal fulfillment in these influential theoretical perspectives not only reflects, but also serves to legitimize neoliberalism and associated selfways” (Adams et al., 2019, p. 204). According to Adams et al. (2019), positive psychology portrays the self as an ongoing development project and is rooted in the idea that personal growth promotes individual flourishing. By championing individual growth and affective regulation as the key to optimal well-being, positive psychology and its growing importance have served to reproduce and reinforce the influence and authority of neoliberal systems. From this perspective, the spread of positive psychology and its expressions in positive education programs around the world represents a form of hegemonic cultural influence. Studying how teachers in non-Western contexts (e.g., Hong Kong) respond to the inflow of positive education programs in their school may provide insights on when local people accept or reject cultural influence from the West.

Cultural psychology has made important contributions to the understanding of why cultural insiders display culturally typical behaviors (see Cohen and Kitayama, 2019). Research has systematically examined how institutions, perceived descriptive norms and personal preferences constrain human psychology, creating systematic cultural differences in behaviors. However, how the same pathways of cultural influence affect the way people evaluate imported practices has received relatively little empirical attention. To fill this gap, the present study examined how Hong Kong teachers evaluated positive education programs, education programs imported to Hong Kong from North America and Australia.

In the following sections, we will first review the three pathways to cultural influence that have been systematically researched. Next, we will present the context of the present investigation. Finally, drawing on the strong situation hypothesis (Cooper and Withey, 2009) and the theory of intersubjective culture (Wan et al., 2007a, b), we develop two hypotheses regarding the circumstances under which personal preferences would have appreciable impact on the evaluation of imported practices.

## Pathways of Cultural Influences

Cultural psychologists, in their attempts to explain why people display culturally typical judgments, have identified three pathways of cultural influence on judgment. First, culture can influence judgment through personal preferences (i.e., internalized values and beliefs). When individuals embrace and identify with the underlying beliefs or values a certain practice embodies, they will judge the practice favorably (Schwartz and Bilsky, 1990). As an example, consider an American teacher’s evaluative responses to a positive education program in their school. Positive education is a movement that aims to promote students’ learning outcomes as well as psychological wellbeing by applying positive psychology theories in education practices (Seligman and Adler, 2018). A positive psychology construct that has been used extensively in the design of positive education programs is the growth mindset – the belief that students can improve their abilities by mobilizing effective effort (Dweck, 2013). A teacher who possesses a growth mindset is expected to evaluate positive education positively.

However, recent research has questioned the efficacy of personal preferences in explaining why people display culturally typical behaviors. For example, in individualist societies, people who display some individualist behaviors (e.g., cognitive dissonance, socially disengaged emotions) do not have a greater tendency to display other individualist behaviors (e.g., field independence, analytical thinking; Na et al., 2010). This result raises the issue of whether people’s behaviors are coherently organized around widely shared preferences for individualism in individualist societies. In addition, measures of individual differences in self-construal and individualism-collectivism do not always mediate cross-differences in behaviors that are relevant to these cultural constructs (see Chiu et al., 2010). Some researchers have argued that personal preferences impact private attitudes more than they do public behaviors (Fischer, 2006), although stronger effects of perceived norms vs. personal preferences have been observed on private cognitions as well (e.g., attribution; Zou et al., 2009). Another view is that personal preferences weigh more heavily than perceived norms in individualist societies than in collectivist ones (Cialdini et al., 1999), although extensive evidence for stronger effect of perceived norms vs. personal preferences on judgment has been reported in both individualist and collectivist cultures (Zou et al., 2009). One explanation for these results is that personal preferences are unimportant predictors of the likelihood that individuals will display culturally typical behaviors. An alternative explanation is that personal preferences influence behaviors, although their effects are often circumvented by normative factors (see Savani et al., 2015). Thus, instead of evaluating the size of the main effect of personal preference, it is prudent to consider under what normative circumstances personal preference will have appreciable effect on judgment.

Culture can influence judgment through institutionalized practices. When a certain practice is a part of the institution in an organization, the organization has set up physical support, as well as formal mechanisms for coordinating and assessing the effectiveness of the practice (Kwan and Chiu, 2015). The fish is the last to discover water. When a certain practice has become part of an organization’s institution, people in the organization tend to align their judgment with the organization, evaluating the institutionalized practice favorably, even when they are not aware of the institutional influence of culture (Na et al., 2010). In the positive education example above, teachers will evaluate the positive education programs in their schools favorably if the school has already provided strong institutional support for it – material support is provided to the teachers practicing positive education; champion teachers have been appointed to coordinate the practice of positive education in the school; and mechanisms have been set up to assess and improve the effectiveness of the positive education programs.

Finally, culture can influence judgment through perceived descriptive norms (Morris et al., 2015). Perceived descriptive norms refer to the values and beliefs that are expected to be popular among members in the organization (Chiu et al., 2010). When individuals perceive that most of their peers embrace the beliefs or values behind a certain practice, even when the individuals themselves did not identify with these beliefs or values, they may still align their judgment with the perceived norms (Yamagishi et al., 2008; Zou et al., 2009). Again, consider the positive education example. Some teachers may not subscribe to the belief in malleable abilities. However, when they expect most teachers in the school to embrace the growth mindset, they may also judge positive education favorably out of conformity to the perceived descriptive norms.

## Cultural Influences Overlap and Reinforce One Another

Although these three pathways (personal preferences, institutionalization, perceived norms) are sometimes portrayed as competing explanations of culturally typical behaviors (Yamagishi et al., 2008; Zou et al., 2009), these sources of cultural influences are not mutually exclusive. Instead, they have partial overlaps, and they reinforce one another (Leung and Morris, 2014). For example, an organization that has (vs. has not) provided institutional support to a certain practice tend to have more members who embrace the values and beliefs behind the practice and perceive these values and beliefs to be widely shared among other members in the organization. Likewise, an organization with more members embracing the values and beliefs behind a certain practice also tends to have perceived norms that are congruent with these values and beliefs. We tested this contention in the current study.

## The Context of Positive Education in Hong Kong

As mentioned at the outset, how the three pathways of cultural influence affect how an imported practice is evaluated has received relatively little empirical attention. To fill this gap, we studied how teachers in Hong Kong evaluated imported positive education programs in their schools.

The positive education movement received primary inspirations from positive psychology. According to Seligman and Adler (2018), “The goal of PE (Positive Education) is to produce both well-being as well as to forward the traditional outcomes of schooling” (p. 54). To attain this goal, educators have designed positive education programs to produce visible wellbeing for both teachers and students by incorporating various evidence-based positive psychology theories in teaching. In a recent comprehensive review of evidence-based positive education programs around the globe, Seligman and Adler (2018) list the following positive psychological theories or constructs that have been incorporated in positive education programs and their respective evidence strength: self-perceptions (growth mindset, self-efficacy; medium evidence strength); achievement theories (achievement goal, intrinsic motivation, value-expectancy theory; medium to high evidence strength); perseverance (grit, engagement; low evidence strength); self-control (medium evidence strength); metacognition (high evidence strength); social competencies (leadership and social skills; evidence strength varied across specific skills); resilience and coping (medium evidence strength); and creativity (low evidence strength).

We use teachers’ responses to positive education programs *in Hong Kong* as a case study to examine when personal preferences predict favorable evaluation of imported positive education practices for two reasons. First, positive education developed from the intellectual tradition of positive psychology in North America. Tahler (2019) has reviewed the intellectual history of positive psychology. According to her review, five major influencers of psychology include William James, Abraham Maslow, Martin Seligman, Mihaly Czikszentmihalyi and Christopher Peterson. Some influential constructs or theories in positive psychology include Albert Bandura’s self-efficacy, Donald Clifton’s strength-based psychology, Edward Deci and Richard Ryan’s self-determination theory, Ed Diener’s subjective well-being, Carol Dweck’s growth mindset and Barbara Fredrickson’s theory of positive emotions (Tahler, 2019). All theories or constructs have had significant impact on the design of positive education programs, including those used in the schools in Hong Kong.

Second, positive education is new to Hong Kong. In their review of positive education programs around the world, Seligman and Adler (2018) found that some countries or cities in Asia have introduced positive education programs in their schools. These countries or cities include Bhutan, Shenzhen, Beijing, and India. Hong Kong was not mentioned in the review. Although education in Hong Kong is largely modeled on the English system, the Confucian heritage, which emphasizes educational achievement and prescribes authoritarian teacher-student relationships, still has a strong influence on the school culture and educational practices in the city (Ho et al., 2001). The 2018 PISA study carried out by OECD (2019) revealed that Hong Kong high school students performed much better than their international peers in reading (reading score = 524 for Hong Kong; OECD mean = 487), mathematics (mathematics score = 551 for Hong Kong; OECD mean = 489) and science (science score = 517 for Hong Kong; OECD mean = 489). Despite their superior academic performance, much fewer Hong Kong students were satisfied with their lives (52% for Hong Kong, OECD average = 67%). In addition, much more Hong Kong students reported “always feeling sad” (13% for Hong Kong, OECD average = 6%), and fewer Hong Kong students possessed a growth mindset (43% for Hong Kong, OECD average = 63%). In short, positive education and its primary concepts (e.g., psychological wellbeing and growth mindset) was still a foreign idea in Hong Kong around 2018 when we conducted the present study^1^.

The mental health crisis in schools has provided the motivation to introduce positive education in Hong Kong. Results from a survey of 3000 Hong Kong students carried out in 2016 revealed that 64% of students felt worried or frustrated and more than 50% felt useless (Fung, 2017). Partly as a response to this mental health crisis, some schools in Hong Kong started to practice positive education. Among them, four primary schools and three secondary schools joined the JC-PEAR program in 2017. JC-PEAR is a positive education program led by the Chinese University of Hong Kong and funded by the Hong Kong Jockey Club. Prior to joining this program, these schools had adopted some improvised practices of positive education. After joining the program, teachers in these schools received training in theories of positive psychology and coaching on the design of positive education practices. In early 2018 and 2019, teachers from these schools voluntarily participated in the current study.

To reiterate, the goal of the present study is to examine under what circumstances individuals’ personal preferences predict their evaluation of imported ideas or practices. We used positive education programs in Hong Kong as an example of imported practices, and teachers’ growth mindset as an example of personal preference. Answering our research question in the education context seems appropriate because teachers play an important role in the transmission as well as evolution of cultural values (Schwartz and Bilsky, 1990). They pass values and beliefs from mainstream heritage cultures to the new generations. In addition, teachers integrate ideas and knowledge from different cultures to create new knowledge, disseminate this new knowledge to their students, and create new cultures through education. In the context of the present study, teachers learn ideas about positive psychology from North American-Australian cultures and innovate local practices to create new education cultures in Hong Kong. Growth mindset, the idea that people’s abilities and personality can develop through deliberate practices of effective learning strategies, is a major positive psychology concept in positive education. In contrast, fixed mindset is the belief that people’s abilities and personalities are non-malleable (Dweck, 2013). We can ask under what circumstances teachers’ internalized mindset would have a more pronounced impact on their evaluation of imported positive education programs^2^.

Aside from conferring an opportunity to test our hypotheses regarding when internalized preferences are more predictive of judgment, the current context also enables us to explore when internalization of an idea (growth mindset) in a *foreign* movement (positive education) can predict local acceptance of the movement.

## When Do Personal Preferences Matter?

We submit that personal preferences influence evaluation of an imported practice, although their effects are often circumvented by normative factors. Under what normative circumstances would personal preference have appreciable effect on the evaluation of an imported practice?

The strong situation hypothesis (Cooper and Withey, 2009) states that the impact of personal preferences (internalized values and beliefs) on judgment is accentuated when situational strength is low, and attenuated when it is high. Situational strength is defined as the amount of environmental pressure on the individual to engage in a particular behavior. Situational strength for a certain behavior is high when there is strong institutional support or normative expectation for the behavior. According to this hypothesis, personal preferences on judgment would have significant influence on the evaluation of a practice when the practice has weak institutional support *and* when the values or beliefs behind the practice are not widely shared in the organization. In the present research context, teachers’ mindsets would have strong influence on their evaluation of positive education programs when positive education is not yet perceived to be an institutionalized practice and when growth mindset is not yet perceived to be a descriptive norm in the school. This hypothesis is consistent with the contention that personal preferences have greater effect on behaviors in loose societies (societies in which social norms are flexible and informal) than in tight societies (societies in which social norms are clearly defined and reliably enforced; Gelfand, 2018).

Another hypothesis is based on the intersubjective theory of culture (Chiu et al., 2010). According to this theory, individuals identify with the organization more strongly when their personal preferences are consistent with the perceived descriptive norms in the organization (Wan et al., 2007a, b). If a particular practice is an established one in the organization, these individuals would evaluate the practice more favorably because of the link between the practice and the organization they identify with.

In the present research context, teachers having a growth mindset will identify more strongly with their school if they expect growth mindset to be a widely shared belief among the teachers in the school. If in this school, positive education is an established practice, these teachers would evaluate the positive psychology programs in their school favorably because these programs are parts of the school’s institution. Accordingly, teachers’ mindset would have relatively strong influence on teachers’ evaluation of their school’s positive education programs when the following two conditions are met: (1) teachers’ mindset is congruent with the mindset perceived to be widely shared among other teachers, and (2) positive psychology is perceived to be an established practice in the school.

The above discussion leads to the following hypotheses:

H1: The effect of personal preferences on the judgment of a practice is more salient in weak (vs. strong) situations, where a weak situation is defined as one in which the practice is not an institutionalized one in the organization and the perceived descriptive norms in the organization are unclear.
H2: The effect of personal preferences on the judgment of a practice is more salient when teachers’ preferences are congruent with the perceived descriptive norm *and* when the practice has already been institutionalized in the organization.

In short, we hypothesize that internalization of an idea by the teachers is important both when positive education (an imported movement) is perceived to be an established practice in the organization and when it is not. When it is, alignment of personal preferences with perceived descriptive norms enhances conformity with the perceived institutional norms and predicts evaluation of positive education programs (H2). When it is not, the conformity pressure is weak and personal preferences should predict evaluation of the positive education programs (H1).

## Materials and Methods

### Context

The JC-PEAR positive education program started in August 2017. At the beginning of the program, the school principals informed the teachers that their schools had joined a 3-year positive education program co-organized by the Chinese University of Hong Kong (CUHK) and the Hong Kong Jockey Club. Teachers also learned from the school leadership that the schools aimed to develop a whole-school approach to positive education that would engage all teachers, students and their parents. In the first year of the program, all teachers participated in professional development workshops offered by the CUHK expert team. The workshops introduced teachers to basic concepts in positive psychology (e.g., growth mindset, grit, character strengths). All teachers were encouraged to apply these concepts in their teaching.

Initially, five to six teachers from each school volunteered to be champion teachers. Throughout the program, they received coaching from the CUHK expert team and gradually became proficient in innovating curricula, pedagogies and assessment to promote students’ learning motivation and psychological wellbeing. The champion teachers also proactively disseminated their learning and experiences to other teachers in the school.

### Sample and Recruitment Procedures

As mentioned, the participants were teachers from seven schools: 4 primary and 3 secondary schools. These schools covered a wide range of academic prestige, from least prestigious schools to most prestigious ones. The teachers completed the first survey in June-July 2018 and the second survey in June-July 2019. A total of 253 teachers participated in the first survey in 2018. In 2019, 293 teachers from the same schools participated in the study. Most of them had participated in the 2018 surveys, although we did not know exactly how many teachers participated in both surveys. This is because to protect teachers’ anonymity, we did not record the participants’ personal identity in both waves of data collection. For the same reason, we were unable to match the data from the two waves. Therefore, we treated the year of data collection as a between-participants factor. We acknowledge that treating the year of data collection as a between-participants factor is a limitation in our research design, because it would lead to under-evaluation of the effect of the year of collection.

Table 1 shows the distributions of the teachers across school types (primary or secondary schools), gender, age categories and educational attainment in the two waves of data collection. Also included in the table are descriptive statistics of teachers’ years of teaching and years of teaching in their current schools. As shown in Table 1, there were no discernible differences between the two waves of data collection in these teacher characteristics.

**TABLE 1:** Summary of teacher characteristics in wave 1 and 2.

Teacher characteristics	Wave 1 *N*(%)	Wave 2 *N*(%)	χ ^2^ (*p*-value)
Sample size	253	293	
Gender			0.49 (0.483)
Male	88 (35.8)	88 (32.8)	
Female	158 (64.2)	180 (67.2)	
Type of schools			0.88 (0.347)
Primary schools	144 (56.9)	155 (52.9)	
Secondary schools	109 (43.1)	138 (47.1)	
Age (years)			2.11 (0.549)
≤30	52 (23.1)	47 (18.0)	
31–40	79 (35.1)	102 (39.1)	
41–50	68 (30.2)	80 (30.7)	
>50	26 (11.6)	32 (12.3)	
Education			4.25 (0.120)
Undergraduate degree	132 (53.4)	156 (58.4)	
Postgraduate degree	106 (42.9)	108 (40.4)	
Others	9 (3.6)	3 (1.1)	
**Teacher characteristics**	**Wave 1 *Mean* (*SD*)**	**Wave 2 *Mean* (*SD*)**	***t* (*p*-value)**
Years of teaching	14.06 (8.65)	13.85 (8.12)	0.28 (0.777)
Years of teaching in current school	11.62 (9.50)	10.86 (7.54)	0.99 (0.325)

To recruit the participants, we sent an invitation to participate to the school administrators. The invitation included a QR code that was linked to the survey. The school administrators forwarded the code to the teachers through e-mail or other e-platforms. Teachers were free to choose to participate in the survey or not. We did not know the number of teachers that the school administrators had invited to participate in the study. There were 60–70 teachers in each school. Assuming that the invitation was sent to all teachers, the response rates were at least over 50% in each survey.

### Measures

Aside from the demographic information collected at the end of the survey, the teachers also completed measures of growth and fixed mindsets, perceived endorsement of growth and fixed mindsets among their peers (perceived descriptive norms), perceived institutionalization of positive education, and evaluation of the positive education programs in their school.

#### Growth and Fixed Mindsets

Mindset is a domain-specific construct; individuals who endorse a growth mindset in one domain may endorse a fixed mindset in another domain, and vice versa (Dweck et al., 1995). Thus, we measured growth and fixed mindsets in both the intelligence domain and the personality domain, the two domains that have been most widely researched in the past.

Each mindset was measured with 2 items adopted from Dweck et al. (1995). The items used for measuring growth mindset in the intelligence domain were: “You can substantially change how intelligent you are”; and “No matter who you are, you can significantly change your intelligence level” (Cronbach’s α = 0.78 in the 2018 survey and 0.77 in the 2019 survey). The items used for measuring growth mindset in the personality domain were: “People can always substantially change the kind of person they are”; and “No matter what kind of person someone is, they can always change very much” (Cronbach’s α = 0.85 in the 2018 survey and 0.83 in the 2019 survey). We used the following two items to measure fixed mindset in the intelligence domain: “You have a certain amount of intelligence, and you really can’t do much to change it”; and “Your intelligence is something about you that you can’t change very much” (Cronbach’s α = 0.85 in the 2018 survey and 0.82 in the 2019 survey). Fixed mindset in the personality domain was measured with these two items: “The kind of person someone is something very basic about them and it can’t be changed very much”; and “People can do things differently, but the important parts of who they are can’t really be changed” (Cronbach’s α = 0.74 in the 2018 survey and 0.78 in the 2019 survey). Participants indicated their extent of agreement or disagreement with each item on a 7-point scale, from 1 (strongly disagree) to 7 (strongly agree).

Items measuring mindsets in the intelligence domain are personally phrased (“*You* can substantially change how intelligent *you* are.”), whereas those measuring mindsets in the personality domain were not (“*People* can do things differently, but the important parts of who they are can’t really be changed). This is because researchers have been interested in how intelligence mindsets affect individuals’ responses to their own achievement outcomes and how personality mindsets affect individuals’ responses to other people’s behaviors (see Dweck et al., 1995).

The specific instructions for these items were: “Using the scale below, please indicate the extent to which you agree or disagree with each of the following statements. There are no right or wrong answers. Please answer each of the following questions honestly.”

#### Growth and Fixed Mindset Intersubjective Culture

We used the same mindset items and scale to measure the perceived descriptive norms of growth and fixed mindsets in the two domains. Following the procedures in past studies (Wan et al., 2007a, b; Zou et al., 2009), after the teachers had responded to the items that measured their personal beliefs about intelligence and personality, we asked teachers to estimate how most teachers in their school would respond to each of the 8 items. The instructions of this measure were: “For each of the statements below, which of the answers would *most teachers in your school choose*? If you think most of them would choose “Agree,” then choose that option for that statement. We strongly encourage you to answer all questions. There are no right or wrong answers.”

Reliability (Cronbach’s α) was 0.88 for growth mindset– intelligence in the 2018 survey and 0.83 in the 2019 survey; 0.83 for growth mindset–personality in the 2018 survey and 0.81 in the 2019 survey; 0.79 for fixed mindset–intelligence in the 2018 survey and 0.80 in the 2019 survey; and 0.85 for fixed mindset–personality in the 2018 survey and 0.78 in the 2019 survey.

Although the same items were used in the personal preference measure and the descriptive norm measure, past studies that used the same method had provided evidence for the discriminative validity of the two measures. For example, the descriptive norm measure predicts judgment and behaviors above and beyond the personal preference measure. Furthermore, the interaction of the two measures predict group identification above and beyond the main effects of both measures (Wan et al., 2007a, b; Zou et al., 2009).

#### Perceived Institutionalization of Positive Education

We were not able to find an established measure of perceived institutionalization of positive education. Therefore, we developed one to assess the extent to which the teachers perceived positive education to be an institution in their school. This measure consisted of four items, which captured the extent to which the school had set up physical support, as well as formal mechanisms for coordinating and assessing the effectiveness of positive education activities. These items were “Teachers who are involved in positive education have reduced workload in other job aspects”; “Our school has provided material resources for us to implement activities in the positive education program”; “There is someone in our school responsible for coordinating the use of different positive education strategies”; and “Our school has mechanisms to help colleagues understand their effectiveness in doing positive education^3^.” Participants indicated their agreement with each item on a 7-point scale, from 1 (strongly disagree) to 7 (strongly agree). Reliability of the measure (Cronbach’s α) was 0.85 in the 2018 survey and 0.87 in the 2019 survey.

#### Evaluation of Positive Education

Our dependent variable was teachers’ evaluation of positive education program (PEP). We used a measure Elfrink et al. (2017) developed to assess evaluation of PEP. This measure consists of five items: (1) “PEP is a valuable addition to our school”; (2) “PEP made me look more consciously at the well-being and engagement of the students”; (3) “PEP changed the school climate to a more positive climate”; (4) “PEP improved my relationship with the students”; and (5) “PEP made me become a better teacher”. Participants indicated their agreement with each item on a 5-point scale, from 1 (strongly disagree) to 5 (strongly agree). Reliability of the measure (Cronbach’s α) was 0.90 in the 2018 survey and 0.94 in the 2019 survey.

## Results

### Cluster Analysis of the Mindset Measures

**FIGURE 1:**
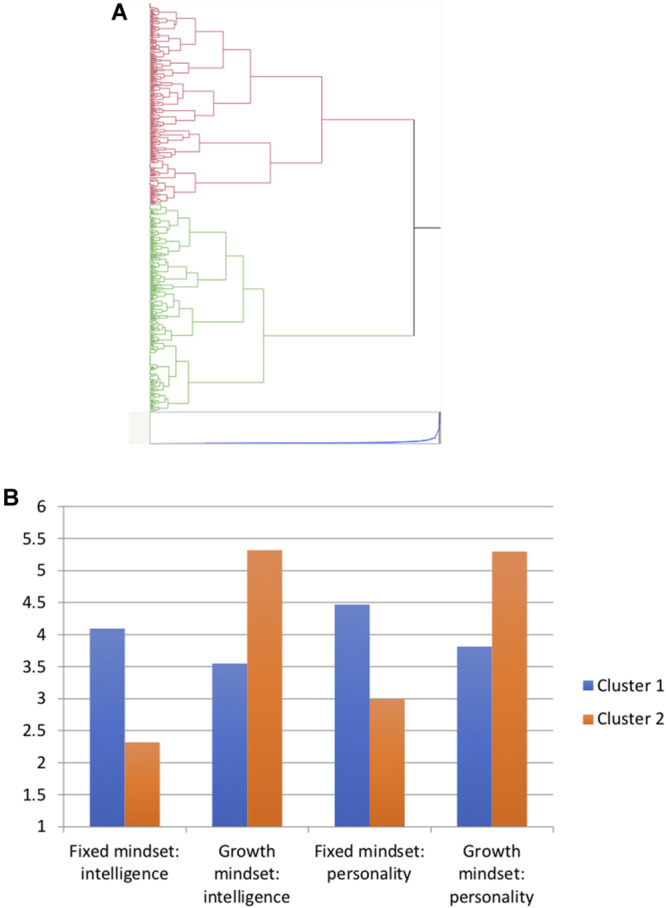
Cluster analysis performed on teachers’ mindset measures: **(A)** A dendrogram representing the cluster analysis results, and **(B)** means of the two clusters on the four mindset measures.

To identify groups of teachers holding different mindset profiles, we performed hierarchical cluster analysis using Ward’s method on the four mindset measures: growth and fixed mindsets in the intelligence and the personality domains. As shown in Figure 1A, two major clusters of teachers were identified. To interpret this result, we performed t-tests on the four mindset measures, with membership in the two clusters as the independent variable. From these analyses, we can identify the mindset measures that differentiated participants in the two clusters. The two clusters differed along all four mindset measures: *t*(543) = −18.28 for intelligence fixed mindset, −13.81 for personality fixed mindset, 18.11 for intelligence growth mindset, and 15.02 for personality growth mindset, all *p*s < 0.0001. Figure 1B shows the means of the two teacher clusters on the four mindset measures. Cluster 1 (*N* = 261, 47.9%), represented by the blue bars and labeled *fixed mindset teachers*, had significantly higher means on the two fixed mindset measures and lower means on the two growth mindset measures, compared to Cluster 2 (*N* = 284; 52.5%, represented by the orange bars and labeled *growth mindset teachers*^4^.

**FIGURE 2:**
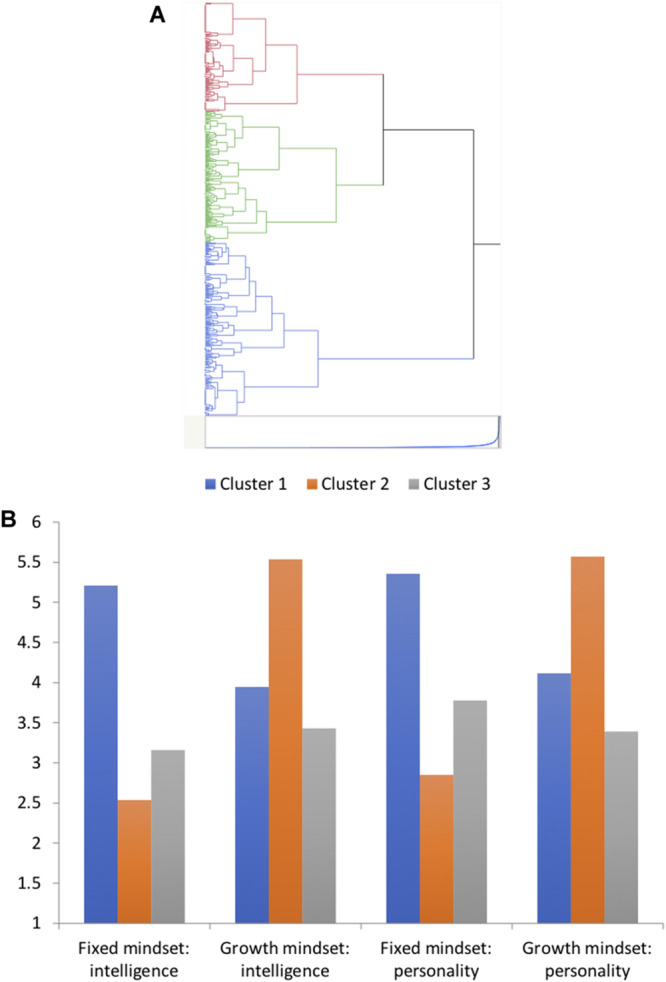
Cluster analysis performed on the four measures of perceived mindset norms: **(A)** A dendrogram representing the cluster analysis results, and **(B)** means of the four clusters on the measures of perceived mindset norms.

We also performed hierarchical cluster analysis using Ward’s method on the four measures of mindset perceived norms. As shown in Figure 2A, three profiles of mindset perceived norms were identified. We performed one-way analysis of variance on each of the four measures of mindset perceived norms, with membership in the three clusters as the independent variable. Tukey’s Honestly Significant Test was used as the method of mean comparisons. These analyses enable us to identify the mindset perceived norm measures that differentiated the three clusters. Significant differences were found among the clusters on all four mindset perceived norm measures: *F*(2, 537) = 476.18 for intelligence fixed mindset, 258.64 for personality fixed mindset, 177.85 for intelligence growth mindset, and 190.36 for personality growth mindset, all *p*s < 0.0001. Figure 2B shows the means of the three profiles on the four measures of mindset perceived norms. Cluster 1 (*N* = 179, 33.1%), represented by the blue bars and labeled *fixed mindset norm*, had significantly higher means on the two measures of fixed mindset perceived norms and lower means on the two measures of growth mindset perceived norms, compared to the other two clusters. Cluster 2 (*N* = 158, 29.3%), represented by the orange bars and labeled *growth mindset norm*, had significantly higher means on the two measures of growth mindset perceived norms and lower means on the two measures of fixed mindset perceived norms, compared to the other two clusters. Cluster 3 (*N* = 203, 37.6%), represented by the gray bars and labeled *no mindset norm*, had relatively low mean scores on all four measures of mindset perceived norms.

### Overlap of Personal Preferences, Perceived Subjective Norms, and Institutionalization

**FIGURE 3:**
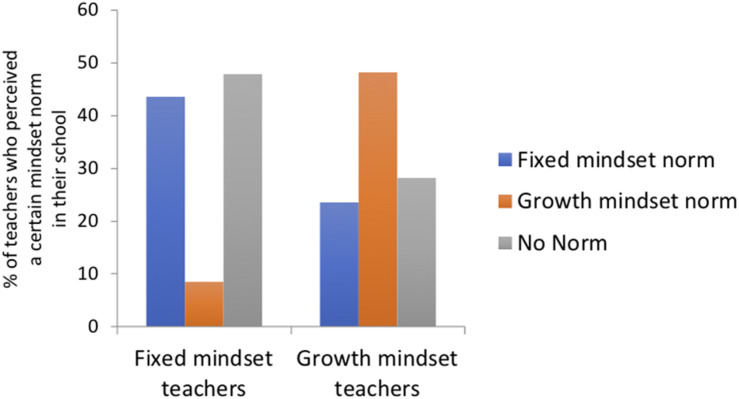
Percentage of teachers who perceived the presence of a growth mindset norm, fixed mindset norm, or no norm in their school among growth mindset teachers and fixed mindset teachers.

We performed logistic regressions to test our assumption that the three pathways to cultural influence are positively associated. For example, did teachers who endorsed a growth mindset also perceived growth mindset to be a descriptive norm in the school? The results are consistent with our assumption. First, logistic regression was performed on the likelihood of belonging to the growth mindset norm cluster (vs. the other two mindset norm clusters) with personal mindset cluster membership as the categorical independent variable. Figure 3 shows that growth mindset teachers were more likely to expect their school to have a growth mindset norm (vs. the other two mindset norms). In contrast, fixed mindset teachers were less likely to expect their school to have a growth mindset norm (vs. the other two mindset norms), χ^2^(*N* = 539, *df* = 2) = 102.99, *p* < 0.0001.

Next, we performed logistic regression on the likelihood of teachers having a growth (vs. fixed) mindset, with perceived institutionalization of positive education as the continuous independent variable. The effect of perceived institutionalization of positive education was significant, χ^2^(*N* = 539, *df* = 1) = 55.51, *p* < 0.0001. To understand the nature of this effect, we estimated the percentage of growth (vs. fixed) mindset teachers when we centered perceived institutionalization of positive education at one standard deviation above (below) the mean. Among teachers who did not perceive positive education to be an institutionalized practice in their school (one standard deviation below the mean), 56.1% were fixed mindset teachers and 43.9% were growth mindset teachers. Among teachers who perceived positive education to be an institutionalized practice in their school (one standard deviation above the mean), 39.8% were fixed mindset teachers and 60.2% were growth mindset teachers.

Finally, we repeated this logistic regression analysis with the likelihood of belonging to the growth mindset norm cluster (vs. the other two norm clusters) as the dependent variable, and perceived institutionalization of positive education as the continuous independent variable. The main effect of perceived institutionalization of positive education was significant, χ^2^(*N* = 539, *df* = 2) = 10.81, *p* = 0.005. Follow-up analysis revealed that among teachers who did not perceive positive education to be an institutionalized practice in their school (one standard deviation below the mean), 22.6% expected other teachers to have a growth mindset, 36.2% expected other teachers to have a fixed mindset, and 41.2% did not expect other teachers to have either a fixed or growth mindset. In contrast, among teachers who perceived positive education to be an institutionalized practice in their school (one standard deviation above the mean), 35.9% expected other teachers to have a growth mindset, 30.1% expected other teachers to have a fixed mindset, and 30.1% did not expect other teachers to have either a fixed or growth mindset.

### When Do Personal Preferences Matter?

To test our hypothesis regarding under what normative conditions personal preferences would predict evaluation of positive education programs, we performed a general linear model analysis on evaluation of positive education programs. The predictors in the model were year of data collection (2018 or 2019), school type (primary or secondary school), teachers’ mindset (fixed or growth), perceived mindset norm (fixed, growth, or no norm) in the school, institutionalization of positive education in the school (mean-centered continuous predictor) and their interactions. We also controlled for the effects of teachers’ gender, age, years of teaching experience, and years of teaching in the current school. The School Type × Year of Data Collection interaction was the only school type effect that approached the 0.05 level of statistical significance (*F* = 3.39, *p* = 0.07; *F*s for all other school type effects < 2.07, *p*s > 0.15). Likewise, with the exception of the School Type × Year of Data Collection interaction, all year of data collection effects were non-significant (*F*s < 1.70, *p*s > 0.18). Therefore, to simplify the model, we removed school type and year of data collection in the subsequent analyses.

The effect of gender was significant, *F*(1, 406) = 4.44, *p* = 0.04. Female teachers evaluated positive education (*M* = 3.86, *SD* = 0.80) slightly more positively than did male teachers (*M* = 3.70; *SD* = 0.78). The effects of age, years of teaching experience, and years of teaching in the current school were not significant (*F*s < 1.17; *p*s > 0.28). In the final model, teachers’ mindset (fixed or growth), perceived mindset norm (fixed, growth, or no norm) in the school, institutionalization of positive education in the school (mean-centered continuous predictor) and their interactions were included as predictors and gender was included as a control variable.

The final model is summarized in Table 2. The effect of gender remained significant, *F*(1, 491) = 5.92, *p* = 0.015. As expected, the main effect of teachers’ mindset (self mindset) was significant, *F*(1, 491) = 24.98, *p* < 0.0001. Growth mindset teachers (*M* = 4.93, *SD* = 1.23) evaluated positive education programs more favorably than fixed mindset teachers did (*M* = 4.53, *SD* = 1.22). We interpret this main effect of personal preference in the context of the significant higher-order interaction reported below.

**TABLE 2:** Summary of the general linear model analysis performed on evaluation of positive education.

Source	DF	SS	F-ratio	*p*-value
Self mindset (self)	1	9.57	24.98	<0.0001
Perceived mindset norm (perceived norm)	2	0.69	0.91	0.41
Institutionalization of positive education (institutionalization)	1	59.52	155.44	<0.0001
Self × perceived norm	2	1.05	1.37	0.25
Self × institutionalization	1	0.05	0.14	0.71
Perceived norm × institutionalization	2	2.56	3.34	0.04
Self × perceived norm × institutionalization	2	3.63	4.73	0.009
Gender	1	2.27	5.92	0.015

The main effect of Institutionalization of Positive Education (Institutionalization) was also significant, *F*(1, 491) = 155.44, *p* < 0.0001. Evaluation of positive education program was more positive when teachers perceived stronger institutionalization of positive education in their school, *r* = 0.77, *p* < 0.0001.

**FIGURE 4:**
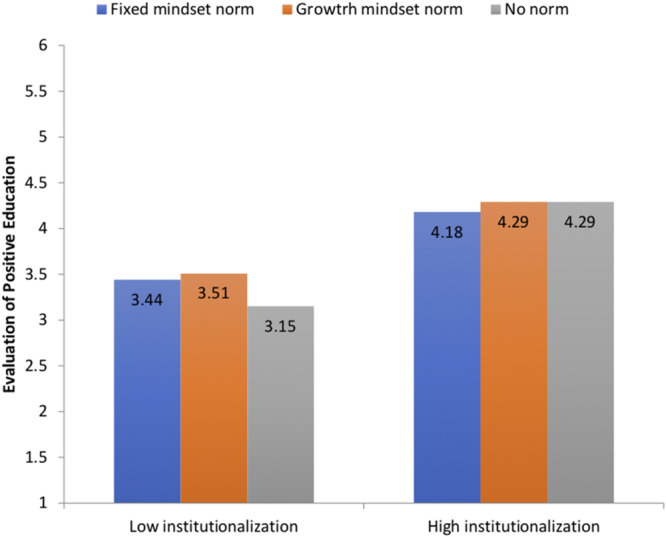
Evaluation of positive education as a function of level of institutionalization of positive education in the school and perceived descriptive norms.

Aside from these two main effects, the Institutionalization × Perceived Mindset Norm interaction was also significant, *F*(2, 491) = 3.34, *p* = 0.04. To understand the nature of the interaction, we centered institutionalization of positive education at one standard deviation both above and below its mean, and estimated the predicted level of evaluation of positive education programs when institutionalization of positive education would be high (one standard deviation above the mean) and low (one standard deviation below the mean) respectively. As shown in Figure 4, when institutionalization of positive education in the school was perceived to be relatively strong (one standard deviation above the mean), positive education programs were evaluated favorably regardless of the perceived mindset norm attributed to the school; estimated evaluation = 4.18 (*SE* = 0.15), 4.29 (*SE* = 0.13), and 4.29 (*SE* = 0.14) for fixed mindset norm, growth mindset norm, and no mindset norm, respectively. However, when institutionalization of positive education in the school was perceived to be relatively weak (one standard deviation below the mean), evaluation of positive education programs was least favorable when teachers did not perceive a prevalent mindset culture in the school; estimated evaluation = 3.15 (*SE* = 0.12), 3.51 (*SE* = 0.16), and 3.44 (*SE* = 0.14) for no mindset norm, growth mindset norm, and fixed mindset norm, respectively. This finding indicates that teachers did not support positive education programs when they did not perceive positive education to be an institutionalized practice and when the descriptive norm was unclear in the school. In short, teachers evaluated positive education most unfavorably in weak situations (no institutional and normative support). This finding underscores the importance of situational support for teachers’ favorable evaluation of positive education programs.

The 3-way interaction is most relevant to our primary research question: Under what conditions would teachers’ mindsets have an effect on the way they evaluated the positive education program? Table 3 illustrates the nature of this three-way interaction. First, the results supported Hypothesis 1, which states that teachers’ mindsets predicted evaluation of positive education programs in weak situations. Specifically, teachers’ own mindsets predicted their evaluation of positive education program only when positive education was not perceived to be an established practice *and* when teachers did not expect other teachers to have a preference for either a growth mindset or a fixed mindset. When positive education was not perceived to be an institutionalized practice (one standard deviation below the mean) and when the teachers did not find any perceived mindset norm in the school, teachers with a growth mindset evaluated positive education programs (estimated value = 3.61, 95% CI: [3.39, 3.83]) more favorably than did those with a fixed mindset (estimated value = 2.95, 95% CI: [2.81, 3.09]).

**TABLE 3:** Estimated evaluation of positive education as a function of institutionalization, self mindset and perceived mindset norm.

	Growth mindset norm	Fixed mindset norm	No norm
**Low institutionalization (1 *SD* Below the mean)**			

Growth mindset (self)	3.54 (3.37, 3.71)	3.63 (3.42, 3.84)	3.61 (3.39, 3.83)
Fixed mindset (self)	3.36 (3.00, 3.72)	3.35 (3.20, 3.51)	2.95 (2.81, 3.09)
Effect of teacher mindset^#^	0.18	0.28	0.66
**High institutionalization (1 *SD* Above the mean)**			
Growth mindset (self)	4.52 (4.30, 4.73)	4.31 (4.18, 4.44)	4.38 (4.19, 4.57)
Fixed mindset (self)	3.96 (3.79, 4.14)	4.09 (3.71, 4.47)	4.19 (4.01, 4.37)
Effect of teacher mindset	0.56	0.22	0.19

**95% confidence interval was indicated in parentheses. ^#^Effect of teacher mindset = Growth mindset – Fixed mindset.**

The results also supported Hypothesis 2, which states that teachers’ mindsets predicted their evaluation of positive education programs when they perceived the institutional and normative contexts aligned with their mindsets. Specifically, when positive education was perceived to be an established practice in the school, teachers with a growth mindset (vs. those with a fixed mindset) evaluated positive education programs more positively when they expected other teachers to have the same mindset. When positive education was perceived to be an institutionalized practice (one standard deviation above the mean), and when the teachers were aware that growth mindset was the perceived descriptive norm in the school, teachers with a growth mindset evaluated positive education more positively (estimated value = 4.52, 95% CI: [4.40, 4.73]) than those with a fixed mindset did (estimated value = 3.96, 95% CI: [3.79, 4.14]).

## Discussion

Most studies in cultural psychology have tried to explain culturally typical behaviors displayed by cultural insiders. Few studies have examined how people judge an imported practice. To fill this gap, the present study examined how Hong Kong teachers evaluated an imported education movement.

Past research on pathways to cultural influences on judgment has compared the explanatory power of personal preferences (internalized beliefs or values), perceived descriptive norms, and institutionalization (see Chiu et al., 2010, 2013). Some past findings show that personal preferences do not always predict culturally typical behaviors by cultural insiders (e.g., Yamagishi et al., 2008; Zou et al., 2009). We show that in our research context, personal preferences predicted teachers’ evaluation of a foreign education movement although the effects of personal preferences were circumvented by normative factors. To elaborate, growth mindset is a positive psychology construct that has been widely used in designing positive education programs (Seligman and Adler, 2018). Accordingly, teachers who subscribed to this belief should evaluate positive education programs more favorably than those who did not. However, this effect was significant only under two circumstances. First, this effect was significant when normative influence was weak – when positive education was not part of the school institution *and* when there were not clear descriptive mindset norms in the school. This result is consistent with the strong situation hypothesis (Cooper and Withey, 2009), which states that effects of personal preferences on behaviors are stronger in weak (vs. strong) situations, in which norms are flexible and informal. Second, this effect was significant when growth mindset was the descriptive norm in the school *and* positive education was part of the school institution. Teachers with growth mindset identified with their school more when they perceived that most teachers in the school also had a growth mindset (Wan et al., 2007a, b). These teachers evaluated positive education programs favorably when they were part of the established institution of the school they identified with. In summary, at least in our research context, in response to an imported practice, personal endorsement of a pertinent belief predicts favorable evaluation of the practice when institution and descriptive norm supporting the practice are *both* weak, *or* when they are *both* strong.

Second, past research on why cultural insiders display culturally typical responses sometimes depicts personal preferences, perceived descriptive norms and institutionalization as competing pathways of cultural influence (e.g., Yamagishi et al., 2008; Zou et al., 2009). In our research context, these three pathways are positively correlated. Teachers who perceived positive education to be an institutionalized practice in their school also tended to endorse a growth mindset (a foundational concept in positive education) and expect other teachers to do so. In addition, teachers who endorsed a growth mindset also expected a growth mindset to be popular among other teachers.

Our findings may not generalize to evaluation of established cultural practices by cultural insiders. Nonetheless, the present study may inspire future research on the normative circumstances under which personal preferences would have significant impact on the likelihood of cultural insiders displaying culturally typical behaviors, instead of pitting the explanatory power of these three pathways against one another.

The present study has limitations. First, to protect the participants’ privacy, we did not collect identifiable information in the surveys. Thus, we could not link participants’ data from the two surveys. The analyses that compared the teachers’ demographics in the two surveys confirmed that we had comparable samples in the two surveys (see Table 1). Nonetheless, given this limitation, we had to treat year of data collection as a between-participants variable in the analysis. This procedure significantly reduced the statistical power of detecting the effects of year of data collection. In addition, we cannot track the participants’ changes in the three predictors (personal mindset, perceived mindset norms, and institutional support) over time and cannot assess the effects of such changes on the evaluation of the positive education programs. We thus missed the opportunity to perform alternative tests of our hypotheses using longitudinal data.

Furthermore, given the correlational nature of our analysis, there are alterative explanations of our results. First, we attribute growth mindset teachers’ relatively favorable evaluation of positive education program in the high institutionalization-strong perceived norm context to their stronger identification with the school. However, it is possible that the strong situation induces compliance and favorable evaluation of the positive education programs among some teachers and psychological reactance and less favorable program evaluation among other teachers. Through the self-perception process or cognitive consistency maintenance, those who evaluate the programs favorably attribute to the self the belief in malleable traits (a belief congruent with positive education). In contrast, those who evaluate the programs unfavorably infer that they believe in fixed traits (a belief incongruent with positive education). Thus, personal preferences do not predict, but instead result from evaluation of the positive education programs.

Second, the strong situation hypothesis assumes that in weak situations, people follow their personal preferences when rendering judgment. However, it is also possible that in weak situations, people infer their personal preferences from their judgment. When there are no clear norms in the school, among teachers who do not receive institutional support for positive education, some would dislike the positive education programs and hence oppose the growth mindset associated with them. In contrast those who like the positive education programs would have insufficient justification for their attitude. To reduce their cognitive dissonance, they would attribute their liking of the programs to their belief in the growth mindset. Our data cannot rule out these and other alternative explanations. Future research is needed to examine them.

We conclude with some practical implications of our results for cultural change. Some organizations or societies may try to create cultural change by importing foreign practices. When a foreign practice is first introduced to a local context, institutional support for the practice is weak and the perceived descriptive norms do not favor the practice or its underlying belief. Under this circumstance, it is important to convert the practice champions into strong believers of the idea behind the practice. These practice champions will perceive the practice favorably and promote it to others. When the practice has been institutionalized and the descriptive norm clearly favors the underlying idea, it is important to solidify the practice in the organization or society by fostering personal identification with the practice among members of the organization or society. These recommendations are applicable when a foreign education practice is introduced to a local school (e.g., when positive education programs are introduced to a local school in Hong Kong).

## Data Availability Statement

The datasets generated for this study are available on request to the corresponding author.

## Ethics Statement

The studies involving human participants were reviewed and approved by Survey and Behavioral Research Ethics Committee, the Chinese University of Hong Kong. The patients/participants provided their written informed consent to participate in this study.

## Author Contributions

All authors contributed equally to the research reported in the manuscript.

## Conflict of Interest

The authors declare that the research was conducted in the absence of any commercial or financial relationships that could be construed as a potential conflict of interest.

## Footnotes

1 The PISA data also showed that the percent of students with a growth mindset was higher in other Chinese societies (Mainland China, Singapore, Taiwan) than in Hong Kong. Therefore, the relatively low prevalence of growth mindset may be unique to Hong Kong’s performance-oriented culture, and does not generalize to Chinese students in general (see Zhang et al., 2019).

2 We do not claim that positive education is effective or culturally appropriate in Hong Kong. Readers who are interested in the efficacy of growth mindset and positive education may refer to recent reviews, empirical research and critical reflections on these topics Seligman and Adler, 2018; OECD, 2019; Rissanen et al., 2019; Yeager et al., 2019).

3 The first two items were not included in the first wave of data collection in several schools. In the 2019 survey, all four items were included in the survey conducted in all seven schools. Effects of the year of data collection when it was included as a moderation in our prediction models were not significant.

4 Because of the lack of comparable teacher data (cf. Laine et al., 2016), we cannot comment on the prevalence of the growth mindset among teachers in Hong Kong, relative to that in other cultures.
